# Mandible-Powered Escape Jumps in Trap-Jaw Ants Increase Survival Rates during Predator-Prey Encounters

**DOI:** 10.1371/journal.pone.0124871

**Published:** 2015-05-13

**Authors:** Fredrick J. Larabee, Andrew V. Suarez

**Affiliations:** 1 Department of Entomology, University of Illinois, Urbana-Champaign, Urbana, Illinois, United States of America; 2 Department of Entomology, Smithsonian Institution, Washington, D.C., United States of America; 3 Department of Animal Biology, University of Illinois, Urbana-Champaign, Urbana, Illinois, United States of America; Arizona State University, UNITED STATES

## Abstract

Animals use a variety of escape mechanisms to increase the probability of surviving predatory attacks. Antipredator defenses can be elaborate, making their evolutionary origin unclear. Trap-jaw ants are known for their rapid and powerful predatory mandible strikes, and some species have been observed to direct those strikes at the substrate, thereby launching themselves into the air away from a potential threat. This potential escape mechanism has never been examined in a natural context. We studied the use of mandible-powered jumping in *Odontomachus brunneus* during their interactions with a common ant predator: pit-building antlions. We observed that while trap-jaw ant workers escaped from antlion pits by running in about half of interactions, in 15% of interactions they escaped by mandible-powered jumping. To test whether escape jumps improved individual survival, we experimentally prevented workers from jumping and measured their escape rate. Workers with unrestrained mandibles escaped from antlion pits significantly more frequently than workers with restrained mandibles. Our results indicate that some trap-jaw ant species can use mandible-powered jumps to escape from common predators. These results also provide a charismatic example of evolutionary co-option, where a trait that evolved for one function (predation) has been co-opted for another (defense).

## Introduction

The significance of predation in animal evolution is reflected in the diversity of strategies that prey employ to avoid it. Antipredator adaptations, such as crypsis, mimicry, chemical defense, or escape behaviors, each derail a specific stage of the predation sequence [1, 2]. Escape jumps and defensive maneuvers, for example, increase the likelihood of prey survival after a predatory attack has been initiated using some combination of rapid acceleration and unpredictable movement. Examples of escape behaviors and protean defenses include some of the most dramatic and metabolically demanding animal behaviors, such as escape jumps in grasshoppers and springtails [3, 4], fast-start swimming in fish [5], and the erratic flights of moths [6]. One of the challenges of studying antipredator strategies, as well as any adaptive trait, is demonstrating that a given behavior is actually used in a predator-prey interaction and results in increased prey survival. Here we present observational and experimental evidence that trap-jaw ants escape from predators using mandible-powered jumps.

Trap-jaw ants have specialized spring-loaded mandibles that snap shut at some of the fastest speeds ever recorded for an animal movement. Members of the genus *Odontomachus*, for example, are capable of snapping their mandibles shut as fast as 60 m/s and generate forces over 300 times their body weight [7, 8]. The trap-jaw apparatus has independently evolved at least four times in ants [9, 10] and is used for disabling fast or chemically-defended prey (e.g. springtails and termites) (reviewed in [11]).

Although all trap-jaw ants use their mandible strike for predation, in at least two lineages, this behavior has been co-opted for defense. Nest-guarding workers of *Orectognathus versicolor* and *Odontomachus ruginodis* direct their strikes against invading ants, flinging the attackers away from the nest entrance [12, 13]. During these interactions, the mandible strike occasionally causes the ant itself to be projected into the air. Although this “jaw jumping” has been observed in trap-jaw ants for over a century, it was generally assumed to be a coincidental byproduct of ants striking large or immovable objects [13, 14]. More recently, Patek et al. [7] suggested that mandible-powered jumping in *Odontomachus* might also be an adaptive escape behavior. They described vertical “escape jumps” following mandible strikes that were targeted against the substrate and were accompanied by a characteristic body and leg posture. Spagna et al. [15] demonstrated that four species of *Odontomachus* can perform these escape jumps, and that the behavior is context-dependent, occurring more frequently in the presence of other predatory ant species.

**Fig 1:**
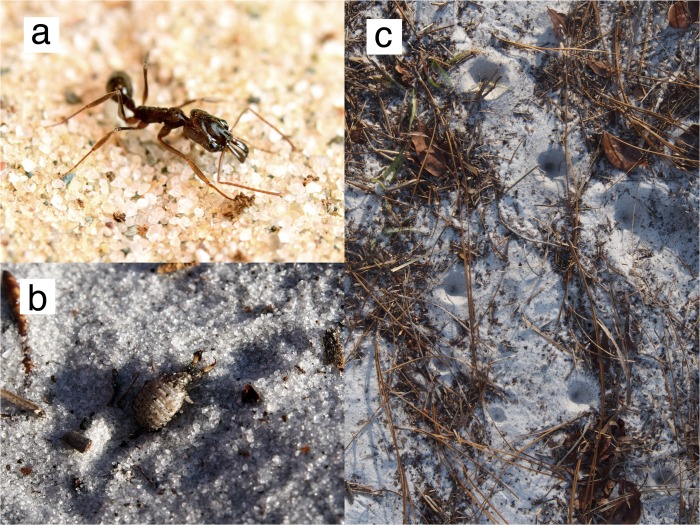
Study organisms. (*A*) a worker of *Odontomachus brunneus* (*B*) a larval antlion, *Myrmeleon* sp., removed from its pit (C) aggregation of antlion pits in sandy soil.

Together, this evidence is suggestive that spring-loaded mandibles have been evolutionary co-opted as an antipredator strategy in some trap-jaw ants, but several fundamental questions about escape jumps remain unanswered. No ecologically relevant predators or competitors have been identified against which trap-jaw ants use their mandibles for escape, and it is unclear if escape jumps actually improve individual survival. While collecting trap-jaw ants in central Florida, we observed ants foraging in areas inhabited by a common sit-and-wait predator: pit-building antlions (Neuroptera: Myrmeleontidae) (Fig 1). Encounters between trap-jaw ants and antlions involve the ants falling into antlion pits, and the ants using their spring-loaded jaws to jump out of the pits. Based on these observations, we tested the hypothesis that the ballistic jumps of *Odontomachus* trap-jaw ants are an effective antipredator escape behaviors by examining: (1) the frequency that trap-jaw ants use escape jumps during encounters with predators that co-occur in their foraging habitat, and (2) if escape jumps increase the probability of a trap-jaw ant surviving a predatory encounter.

## Material and Methods

### Study Site and Species

All field research was conducted at Archbold Biological Station (ABS) with their permission over three summers (2011 to 2013). Twenty nests of *Odontomachus brunneus* were excavated from scrub habitat and pastures at ABS and the MacArthur Agro-ecology Research Center (MAERC), both located at the southern end of the Lake Wales Ridge in central Florida. Colonies ranged in size from approximately 15 to 75 workers, and four contained queens. In the laboratory, artificial nests were constructed from 32 cm X 20 cm plastic boxes with Fluon-coated walls. One or two plastic test tubes containing water trapped behind cotton plugs were provided for nest chambers. Ants were provided with water and 20% sugar solution *ad libitum*, and 4–10 termites or frozen crickets each day.

Pit-building antlions (Neuroptera: Myrmeleontidae) are ubiquitous sit-and-wait predators of terrestrial insects, and are commonly found in trap-jaw ant foraging areas at ABS and MAERC [16] (Fig 1 and S1 Video). The larvae dig conical pit traps in sand or fine soil and lie at the bottom for ground-foraging arthropod prey to fall in [17]. The walls of the pit are unstable and cause would-be escapees to stumble toward the waiting antlion [18]. Antlions also throw sand directly at potential prey items, further destabilizing them [19]. At the bottom of the pit, the antlion grabs prey with long hollow mandibles, pulls them under the sand, and injects enteric fluids into the prey’s body cavity [17, 20]. Where they occur, antlions are a significant ant predator and can even influence the community structure and foraging behavior of ants [21]. Their high population density and amenability to laboratory environments make antlions an ideal ant predator to study predator-prey interactions with trap-jaw ants.

Ultimate (third) instar larvae of *Myrmeleon carolinus*, *M*. *crudelis*, or *M*. *mobilis* were collected along fire lanes and under shelters at ABS. Each antlion was transferred to an arena made from 11.5 cm diameter plastic cups coated with Fluon (Northern Products) and filled with 4 cm of sand collected from the sites where the antlions were found. Sand was sifted with a #20 US standard sieve (Hogentogler & Co., Inc.) to remove large particles. After digging a pit in an arena (usually within 24 hours), antlions were fed one ant (*Dorymyrmex bureni)* each day. Both ants and antlions were maintained at approximately 24**°** C with natural lighting cycles. Vouchers have been deposited in the invertebrate collection at ABS.

### Behavioral Observations

We conducted 117 trials, each consisting of a single encounter between an individual trap-jaw ant and an antlion. The antlions were starved for 48 hours prior to observation and any larvae that had abandoned their initial pit or had not dug a pit at all were excluded from study. Ant workers were allowed to rest individually in Fluon-coated cups for approximately one minute and then tipped into the arena away from the antlion pit. Ants that fell directly into a pit or snapped their mandibles before entering a pit on their own were immediately returned to their nest for later use. Each trial was recorded with either standard (Panasomic TM80 high-definition video camera) or high-speed video (Phantom Miro Ex4 color high-speed camera, 700 frames per second, or Photron 512-PCI camera, 500 frames per second).

A trial started after introducing the ant into the antlion arena and concluded after 10 minutes or when one of the following events was observed: *caught*—the ant was grabbed by the antlion and held under the sand for at least 60 seconds, *escape by running*—the ant ultimately escaped by running out of the pit, and *escape jump*—the ant jumped completely out of the pit as a result of a mandible strike. We only scored outcomes if the antlion attacked (either threw sand at or attempted to grab the ant), and if no interaction occurred within 10 minutes, the trial was terminated and the individuals omitted from the analysis. Pit diameter, which is correlated to antlion size [17], was measured immediately prior to each trial. Ants were frozen following the trial for morphometric analysis. To test the effect of size on the trial outcomes, we used a logistic regression analysis with escape frequency or jump frequency as a binomial response variable, and ant mass and antlion pit diameter as main effects. All statistical tests were implemented in R 3.1.0 [22].

### Restraint Experiment

**Fig 2:**
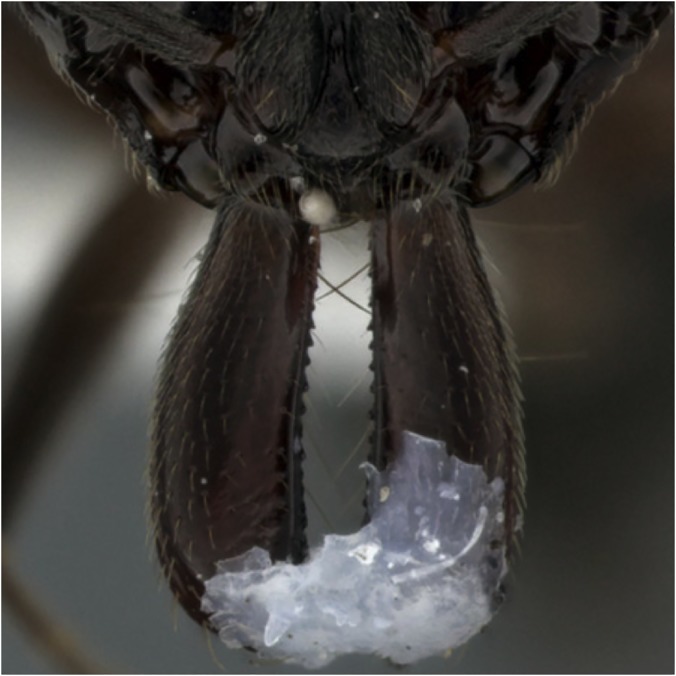
Restrained mandibles. Trap-jaw ant mandibles were glued shut to prevent ants from snapping.

To test the hypothesis that escape jumps improve the survival of individual ant workers, we experimentally prevented workers from jaw-jumping by restraining their mandibles and then measuring their escape frequency from antlion pits. Individual foragers were chilled on ice and the distal tips of the mandibles were glued together with cyanoacrylate glue (Scotch Super Glue Gel) (Fig 2.). After 24 hours of recovery, we tested the escape ability of the ants with the same trial protocol described above. In addition to the experimental restraint treatment, two controls were also included: a mock treatment, where glue was applied only to the outside edge of the mandible, allowing the ant to snap their jaws, and a treatment control, where the ant was not manipulated. Each of the three treatments included 76 replicates. Escape frequency data were entered into a contingency table and analyzed with a log likelihood ratio statistic (G-test).

## Results

**Fig 3:**
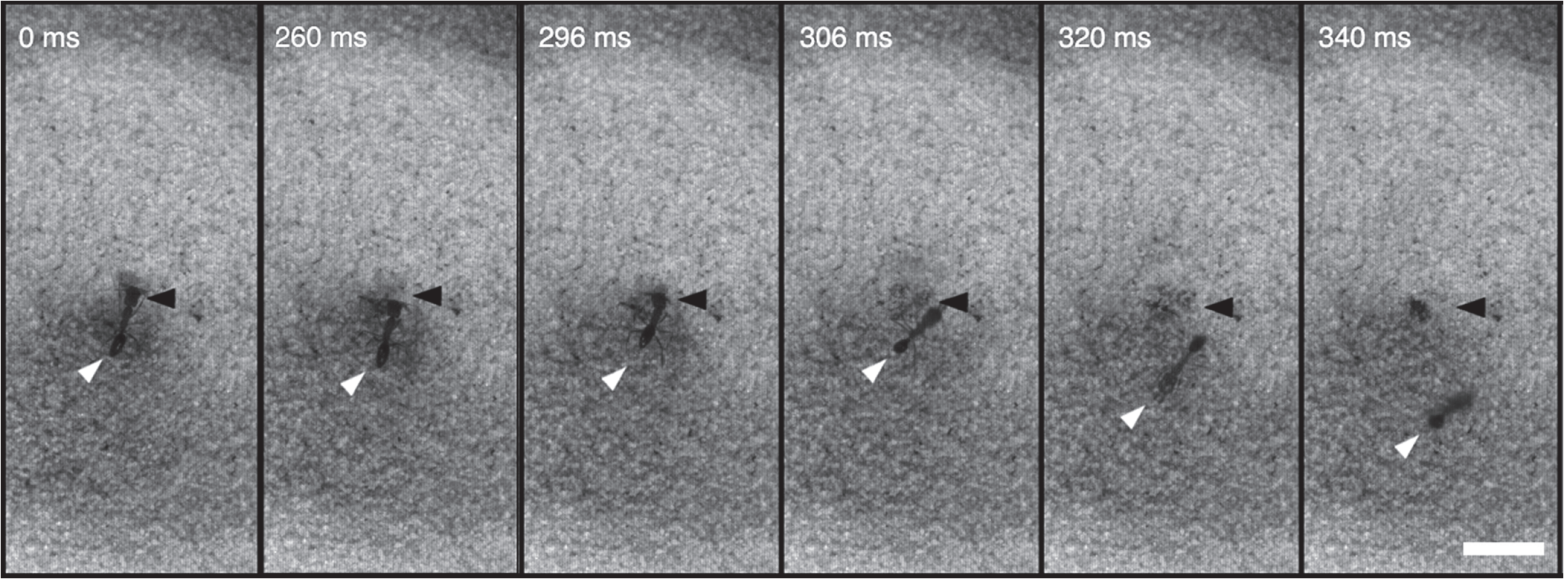
Still images from high-speed video (S2 Video) showing escape jumps of *Odontomachus brunneus* during interactions with the antlion, *Myrmeleon carolinus*. After being attacked by an antlion (location at base of pit marked by black arrows in each frame), the trap-jaw ant (white arrows in each frame) strikes the side or center of the pit with her mandibles and propels herself away from the antlion. Time stamp indicates the time elapsed since first frame (upper left). Scale bar = 1cm.

When *Odontomachus brunneus* workers fell into antlion pits, they were captured approximately one-third of the time (S1 Video). In 15% of the trials, they escaped by jumping with their mandibles, and in the remaining trials they ran out of the pit (Table 1). Ant size, antlion pit diameter, and the interaction between those two variables had no effect on jump frequency (ant size: Wald statistic = 0.599, *P* = 0.549; pit diameter: Wald statistic = 0.736, *P* = 0.462; ant size X pit diameter: Wald statistic = -0.699, *P* = 0.485) or overall escape frequency (pooled escape outcomes by running and jumping: ant size: Wald statistic = -0.256, *P* = 0.798, pit diameter: Wald statistic = -0.411, *P* = 0.681; ant size X pit diameter: Wald statistic = 0.325, *P* = 0.745). The duration of each trial ranged between 0.5 and 65 seconds and did not vary among the three outcomes (Kruskal-Wallis test: H = 4.10, *P* = 0.129). Individuals that escaped by jumping responded to antlion attacks by snapping their mandibles against the side or bottom of the pit, resulting in an explosion of sandy substrate and launching them completely out of the pit (Fig 3 and S1 and S2 Videos). Escape jumps always occurred after an antlion attack. Prior to the strike, the ants exhibited behaviors similar to those previously described for escape jumps [7], including orienting their mandibles and antennae against the substrate and sometimes lifting a leg vertically.

**Table 1:** Summary of Predator-Prey Interactions Between Trap-jaw Ants and Antlions.

	Trial Outcome		
	Caught	Escape via	
		Run	Jump
**Frequency (%)**	36.8	48.7	14.5
**Ant mass (mg)**	7.48 (1.4)	7.42 (1.06)	7.66 (1.37)
**Pit diameter (cm)**	5.3 (1.2)	5.2 (1.2)	5.1 (1.4)
**Length of Trial (sec)**	8.71 (12.9)	7.01 (5.31)	12.12 (8.14)

Based on 117 trials between *Odontomachus brunneus* trap-jaw ants and *Myrmeleon* spp. antlions. Outcome frequencies were not distributed equally (χ^2^ = 21.1, df = 2, *P* < 0.001). All means are provided with standard deviation (in parentheses).

Trap-jaw snaps did not always result in successful escape jumps. In fact, only 26.5% of observed jaw strikes generated jumps. The trajectory of three of these jumps caused the ant to collide with the side of the pit and fall back to the center. Strikes were also sometimes released without targeting after they were grabbed by an antlion, usually missing the antlion or pit. In six cases strikes occurred after the antlion pulled the ant under the sand (S4 Video). Untargeted strikes were sometimes followed by the ant being released by the antlion and subsequently escaping from the pit by running.

**Fig 4:**
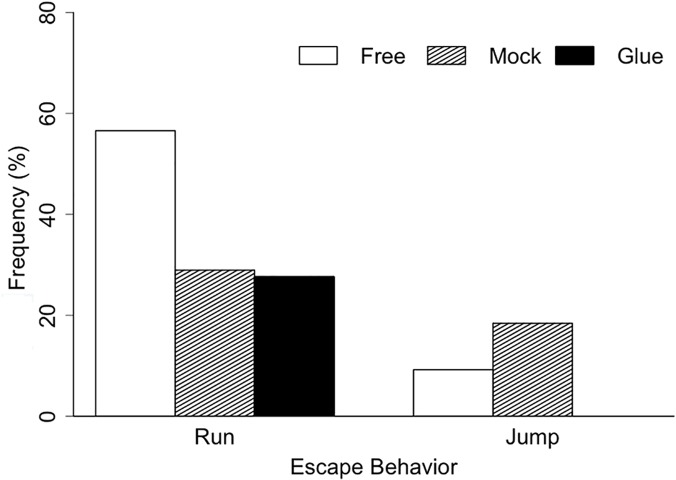
Frequency of escape behavior by trap-jaw ants in restraint experiment. Frequency of escape by running or jumping was quantified for each interaction. Ants were unmanipulated (Control), had glue applied to the exterior edge of mandible (Mock), or had their mandibles glued shut (Restrained). Each treatment was replicated 76 times.

Restraining the mandibles of trap-jaw ants significantly decreased their ability to escape from antlion pits (Table 2). Ants in the mock treatment and unrestrained control were 2.3 and 4.7 times, respectively, more likely to escape than the restraint treatment. Experimental manipulation (chilling and applying glue) reduced the escape success of individuals in the mock and restraint treatment by decreasing their ability to run out of pits (Fig 4). However, manipulation did not affect escape jump frequency of the mock treatment, and the increased survival of ants in this treatment compared with the experimental restraint treatment was due to their use of mandible strikes for jumping (Fig 4).

**Table 2:** Escape Success In Restraint Experiment.

	Treatment			
Trial Outcome	Control	Mock	Restraint	Total
Escape	49	36	21	106
Caught	27	40	55	122
Escape Frequency (%)	64.5	47.4	27.6	
Odds Ratio (95% CI)				
vs. Control	1	–	–	
vs. Mock	2.0 (1.0–3.9)	1	–	
vs. Restraint	4.7 (2.4–9.5)	2.3 (1.2–4.7)	1	

Trial outcome was not independent of treatment (*G*-test *G* ^2^ = 21.3, df = 2, *P* < 0.001, n = 76 per treatment). Odds ratios and 95% confidence intervals (in parentheses) were calculated from partial contingency tables consisting of pairs of treatments.

## Discussion

We observed workers of *Odontomachus brunneus* using their spring-loaded mandibles to escape from the pit traps of antlion larvae and experimentally demonstrated that this behavior increases individual survival of the ants during predatory encounters by about twofold. These results support the idea that in the genus *Odontomachus*, predatory trap-jaws have been evolutionary coopted for defense.

Selection on individual forager survival is likely stronger in *Odontomachus* than in social insects with large colonies of workers where foragers are largely “disposable”. Colonies of *Odontomachus brunneus* typically consist of a maximum of about 100 workers with a high percentage of the colony engaged in foraging activities [23]. Hart and Tschinkel [23] found foragers located throughout the nest, suggesting that foragers may be engaging in critical nest activities other than just food acquisition. Also, unlike many other ants that recruit nestmates to resources [24], trap-jaw ants forage solitarily [21, 25, 26]. Without the protection of large groups of nestmates, trap-jaw ant foragers may be at a higher risk of predation. Escape jumps and other antipredator behaviors that increase forager survival may represent a significant advantage for the colony.

Jumping is probably a particularly successful strategy for escaping from ambush predators like antlions, who rely on the instability of their pit to hinder the fleeing attempts of ants [18]. By jumping, trap-jaw ants literally bypass this tactic and increase their probability of survival. Most animal prey escape behaviors that have been documented are used against active hunting predators such as nursery web spiders [27], odonate naiads [28], and copepods [29]. It is still unknown how effective trap-jaw ant escape jumps are against mobile predators.

Preliminary data with other species of *Odontomachus* suggest that not all trap-jaw ants use their mandibles to escape from antlions. For example, the smaller *O*. *relictus*, which is found in sympatry with *O*. *brunneus* and several species of antlions, does not jump after falling into antlion pits (Larabee and Suarez unpublished data). It is unlikely that *O*. *relictus* workers are incapable of jumping due to physical constraints; the mean maximal force of *O*. *relictus* mandible snaps is similar to those of other *Odontomachus* species that also perform escape jumps [8, 15]. It is unclear if escape jumps are missing from the behavioral repertoire of *O*. *relictus*, or if they require a specific context in which the behavior would be released.

Antlions are only one of numerous predators and competitors with which *O*. *brunneus* interacts. Several other predators have been described that may be specialists on trap-jaw ants, including workers of *Dorymyrmex bureni*, *Formica archboldi* [30], and *Pachycondyla* spp. [26]. Workers of *O*. *brunneus* and *O*. *relictus* were rare in stomach content surveys of the narrow-mouthed toad, *Gastrophryne carolinensis*, an ant specialist whose foraging areas and daily activity overlaps with *Odontomachus* at ABS [31]. The absence of trap-jaw ants from the diet of this ant predator may be due to some anti-predator defense, either its well-developed sting or use of escape jump.

Spring-loaded mandibles may represent a “key morphological innovation”, allowing trap-jaw ants to access an otherwise inaccessible resource (fast and chemically defended species) [32]. In at least some species of the genus *Odontomachus*, however, trap jaws appear to have been evolutionary coopted and play a role on both sides of predator-prey evolutionary arms races. Many questions remain about the evolutionary history of trap jaws. There are more than 160 species in the sister trap-jaw ant genera *Odontomachus* and *Anochetus*, and only a small fraction have been studied in any detail with respect to their defensive use of the mandible snap. A full understanding of the evolutionary dynamics trap-jaw mandibles will require a more comprehensive documentation of the natural history of trap-jaw ants.

## Supporting Information

S1 Video Antlion predatory behavior. This high-speed video (700 fps) depicts a successful antlion predation event. After flinging sand at prey, the antlion grabs an ant and drags it under the sand at the bottom of the pit.
(MP4)

S2 Video Trap-jaw escape jump. This high-speed video (500 fps) depicts an escape jump of *Odontomachus brunneus* during interactions with an antlion, *Myrmeleon carolinus*. After striking the substrate, the ant jumps completely out of the pit.
(MP4)

S3 Video Trap-jaw escape jump. A standard speed (32 fps) video showing the trap-jaw ant *Odontomachus brunneus* jumping out of an experimental antlion pit.
(MP4)

S4 Video Unsuccessful escape jump. This high-speed (500 fps) video shows a trap-jaw ant snapping its jaws after being caught by an antlion, then being released, and an unsuccessful escape jump.
(MP4)
